# Analysis of Physical Features Affecting Glossiness and Roughness Alteration in Image Reproduction and Image Features for Their Recovery

**DOI:** 10.3390/jimaging11040095

**Published:** 2025-03-25

**Authors:** Midori Tanaka, Hideyuki Ajiki, Takahiko Horiuchi

**Affiliations:** 1 Graduate School of Informatics, Chiba University, Yayoi-cho 1-33, Inage-ku, Chiba 263-8522, Japan; horiuchi@faculty.chiba-u.jp; 2 Graduate School of Science and Engineering, Chiba University, Yayoi-cho 1-33, Inage-ku, Chiba 263-8522, Japan; 24wm3216@student.gs.chiba-u.jp

**Keywords:** total appearance, glossiness, roughness, shitsukan management, image reproduction

## Abstract

Digital imaging can cause the perception of an appearance that is different from the real object. This study first confirmed that the glossiness and roughness of reproduced images are altered by directly comparing real and colorimetrically reproduced images (CRIs). Then, psychophysical experiments comparing real and modulated images were performed, and the physical features that influence the alteration of the real object were analyzed. Furthermore, we analyzed the image features to recover the altered glossiness and roughness by image reproduction. In total, 67 samples belonging to 11 material categories, including metals, resins, etc., were used as stimuli. Analysis of the physical surface roughness of real objects showed that the low skewness and high kurtosis of samples were associated with alterations in glossiness and roughness, respectively. It was shown that these can be recovered by modulating the contrast for glossiness and the angular second moment in the gray level co-occurrence matrix for roughness, reproducing perceptually equivalent images. These results suggest that although the glossiness and roughness of real objects and their CRIs are perceived differently, reproducing perceptually equivalent glossiness and roughness may be facilitated by measuring the physical features of real objects and reflecting them in image features.

## 1. Introduction

People judge the quality and behavior of objects based on visual information such as color and total appearance. In recent years, a discussion on total appearance has flourished in the International Commission on Illumination (hereafter CIE), but a method that extends beyond color is needed to manage the total appearance of objects [1,2]. In Japan, “shitsukan”, a concept related to total appearance based on a comprehensive psychological perception derived from physical stimuli, has been actively researched in fields such as information engineering, psychophysics, and neuroscience [3].

Glossiness, which is directly related to an object’s perceived attractiveness and value, is particularly important in appearance design and product development and has been actively studied in psychophysics and neuroscience [4]. For real-world objects, Beuckels et al. [5] attempted to construct a new gloss space by adding object surface haze to the existing gloss space comprising two axes: distinctness-of-image and contrast gloss. Their study suggests that object haze affects perceived glossiness and sharpness. Chen et al. [6] also investigated the display characteristics required to reproduce the glossiness of real objects in images on displays and evaluated the gloss match between real objects and images on HDR stereoscopic displays. The results showed that the luminance and dynamic range of the display affect the gloss match between the real object and the image. In addition, there is active research into methods of reproducing gloss in approaches such as 3D displays and 3D printing [7,8].

Roughness perception is also directly related to the physical surface condition of an object and is known to be influenced by both visual and tactile cues. Ercetin et al. [9] proposed a non-contact method for evaluating surface roughness using image-processing technology, emphasizing the importance of surface quality control in manufacturing. Similarly, in the field of machine vision, roughness is an important metric in high-resolution images, and the improvement and detection of roughness using fractality [10] and image features [11,12] of the object surface have been discussed. The study by Bergmann Tiest et al. [13] also showed the tactile–visual interaction in roughness perception and revealed that physical roughness and perceived roughness do not necessarily match. Therefore, rendering roughness perception in an image requires a technique that equivalently manages the perceived roughness of the real object and the image.

We have researched total appearance management and shitsukan perception using real objects and images for glossiness, transparency/translucency, and roughness perception [14,15], also analyzing the relationship between perceived quality and physical quantities through psychophysical experiments. For example, Tanaka et al. [14] investigated shitsukan perception for real objects and images using 34 real objects comprising 10 materials including stone, paper, glass, etc. and colorimetrically reproduced images (CRIs) with colors equivalent to those of the real objects. The results showed that the perceived qualities of appearance may differ between real objects and CRIs, but there was also a lack of discussion on reproducing an appearance that is perceptually equivalent between images and real objects. For this, a technique is required to make the perceived glossiness equivalent to that of the real object under the physical limitations of the display. Although there have been many studies on glossiness perception [4], the relationship between colorimetry and glossiness perception comparing the real object and the image has not been fully clarified. In addition to comparing the appearance of real and CRIs directly, methods for matching the appearance of the image and the real object must be investigated.

This study focused on reproducing glossiness and roughness in images. Specifically, through psychophysical experiments that directly compare the perceptual quality of two types of appearance (glossiness and roughness) using real objects and CRIs, we confirmed how the differences between them occur and clarified the physical features of the object that influence the difference. Furthermore, we conducted psychophysical experiments to select reproduced images that are perceptually equivalent to the real ones to clarify the image features for perceptually recovering the altered glossiness and roughness.

## 2. Experiment A

This study conducted two psychophysical experiments. Experiment A directly compared the perceived glossiness and roughness of real objects of different materials and their CRIs to verify the perceptual difference between them. Further, the physical features affecting the alteration were analyzed.

### 2.1. Experimental Stimuli

This experiment used 67 real and matching image stimuli comprising 11 different materials: fabric, glass, ceramic, rubber, plastic, metal, wood, leather, paper, stone, and shell.

- Real stimulus

**Figure 1:**
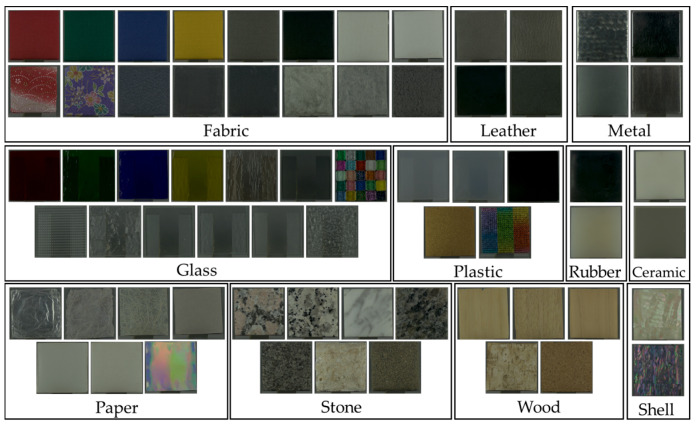
Experimental stimuli. (Note: The sample stand is visible through the glass stimulus, etc., which has the same square pattern on both sides.).

Sixty-seven mundane materials, varying in surface texture, color, and shape but uniform in size, were included (Figure 1). These were in addition to the 34 stimuli used in a previous study [14], with the addition of 33 new stimuli, including two new shell samples. In this study, we randomly selected a mundane sample with a variety of surface irregularities and reflective properties. Figure 1 shows the list of stimuli classified into 11 material categories: 16 fabrics, 13 glasses, 7 papers, 7 stones, 5 plastics, 5 woods, 4 leathers, 4 metals, 2 rubbers, 2 ceramics, and 2 shells. Each real stimulus was a 50 × 50 mm square placed perpendicular to the observer’s sight line and illuminated with a mixture of diffuse and highly directional light equivalent to D65. The light intensity was also adjusted so that the exposure of the observer’s eyes to the real stimuli (specifically, the highlights) did not exceed the maximum luminance of the display (962 cd/m^2^).

- Image stimulus

Image stimuli were generated by photographing the real objects in an observation box with an LED light source equivalent to D65 and reproducing colorimetrically equivalent images. A two-dimensional spectroradiometer (SR-5100, Topcon Technohouse Corp., Tokyo, Japan) was used for the imaging, and the acquired CIE XYZ image data were converted to RGB images using the CIE XYZ to display the RGB conversion matrix created in the prior display calibration and displayed on a calibrated display (ColorEdge CG3146, EIZO Corp., Ishikawa, Japan).

The image stimuli were captured at high resolution, and the pixel size of the stimulus area was 750 × 750 pixels. In addition, an X-Rite ColorChecker was used to verify the color accuracy of the generated CRIs. The tristimulus values of each color patch of the real and CRIs were measured with a spectroradiometer (CS-2000, Konica Minolta, Inc., Tokyo, Japan), and the average color difference for CIE 1976 ΔE*ab was 1.2, which is approximately half the color discrimination threshold. This confirmed that the CRIs were color-reproduced with sufficient accuracy.

### 2.2. Experimental Procedure

In Experiment A, the magnitude estimation (ME) method was used to evaluate the perceived glossiness and roughness of real stimuli and CRIs. The magnitude ME method is a linear rating method in which the rating value of the target is set as a reference value with a positive constant. This value is rated linearly with respect to two indices: 0 and the reference value. In this experiment, the real stimulus was set as the reference with 100 as the criterion, and the rating value was set to be non-negative with no upper limit. For example, when rating the glossiness of the presented image stimulus, observers responded “100” if they thought the glossiness of the image stimulus was the same as that of the real stimulus, and “200” if they thought the glossiness of the image stimulus was twice as high as that of the real stimulus.

**Figure 2:**
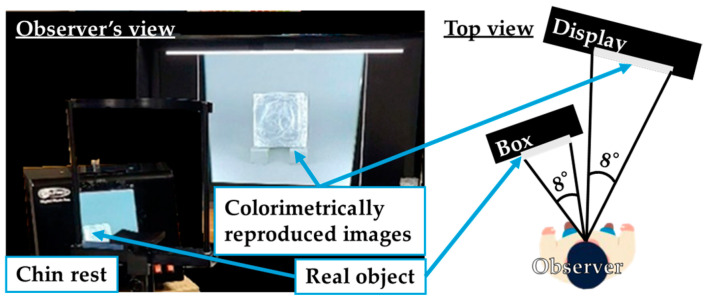
Experimental setup.

The experimental setup is shown in Figure 2. The real stimuli and the CRIs were arranged for an equal 8-degree viewing angle, and the observer could move his gaze to compare them. The observed image resolution was approximately 65 cycles per degree (cpd), which exceeds human resolution at 20/20 acuity (30 cpd), confirming that the image resolution did not affect judgment. This allowed the observer to view the image stimuli with the same light and resolution as the real stimuli.

The observer adjusted the chin rest and adapted to the darkroom for 5 min before beginning the rating experiment. The observer viewed the real stimuli and the image stimuli with both eyes and responded with glossiness and roughness ratings. After being rated, the stimuli were changed, and the process was repeated. All 67 stimuli were rated twice for each observer.

### 2.3. Result of Perceptual Alteration

Ten observers, also in their 20s, participated in Experiments A and B. All observers had previously passed the Ishihara color vision and visual acuity tests and were found to have normal color vision and visual acuity (20/20 or better).

In this experiment, we verified whether the real gloss and roughness perceptions were adequately reproduced in the CRIs. First, the Smirnov–Grubbs test was used to verify whether the value farthest from the average of 10 averages rated twice by each observer was an outlier (*p* < 0.05), and outliers were removed by repeating the test until no outliers were detected. The average of the evaluated values was then calculated. Next, a corresponding *t*-test verified whether the evaluated values of the CRIs changed significantly regarding the glossiness and roughness of the real object, and also confirmed the significance by calculating the effect size d. The effect size indicates the “degree of difference detected” and can be used in conjunction with the *p*-value to determine the statistical significance and the size of the substantial difference [16,17]. In this result, we continue the discussion using the effect size results because the samples with a “medium” or higher effect size, which were found to be significant i.e., d > 0.5, included samples with *p* < 0.05.

**Figure 3:**
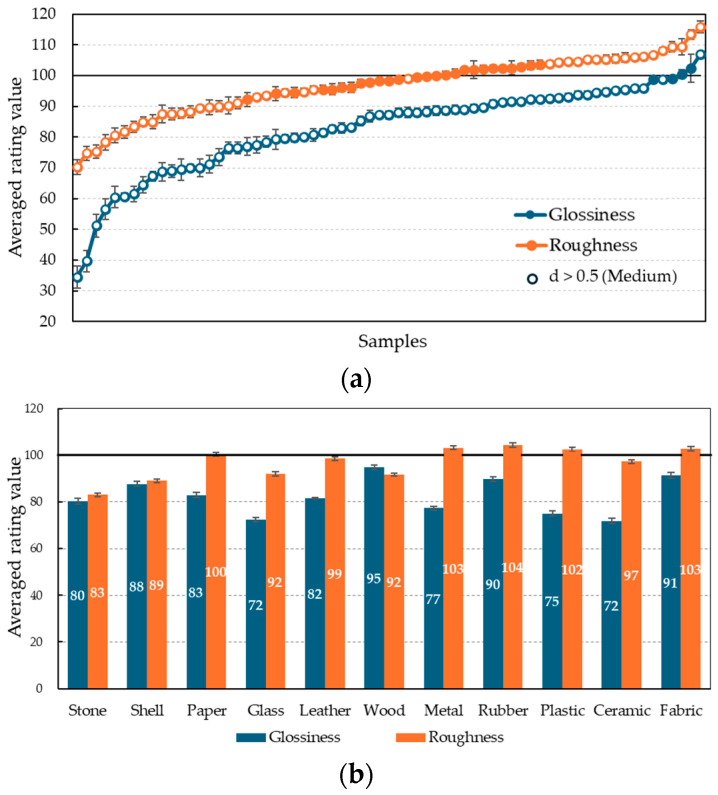
Averaged rating value with standard error for CRIs in Experiment A. (**a**) All samples in ascending order by rating; (**b**) Rating values for material categories.

Figure 3a shows the average rating values for gloss and roughness for each CRI stimulus, with open circles representing samples with valid “medium” or larger (d > 0.5) effect sizes. The average values for each material category are also shown in Figure 3b.

- Glossiness

For 62 of the 67 stimuli, the glossiness of the CRIs was significantly lower (d > 0.5, *p* < 0.05) than the real object (reference value of 100). In particular, a significant decrease was observed for high-gloss materials such as glass, ceramic, plastic, and metal (Figure 3b). Conversely, only one stimulus was rated significantly higher than the gloss of the real stimulus: white canvas (rating for the CRI stimulus: 107.0). This sample is bright white with L* = 97.3 and has a luminance distributed by the fine surface irregularities of the canvas weaves. The white canvas, which is perceived as diffuse in the real object, is perceived as less physically diffuse when reproduced as an image on the display, and thus the variable luminance distribution is more prominent and perceived as glossier.

- Roughness

For 24 of the 67 stimuli, the average roughness rating of the CRI stimuli was significantly lower than that of the real stimuli (reference value of 100), and for 17 stimuli it was significantly higher (d > 0.5, *p* < 0.05). As Figure 3b shows, the samples with significantly lower ratings among the CRIs were mainly in the stone, shell, glass, wood, and ceramic categories, which had relatively smooth or distinctly uneven object surfaces. Conversely, CRI samples with significantly higher ratings, such as fabric and paper, did not have deep surface irregularities on the real objects, but the grains and patterns were emphasized by the imaging and were perceived as visually noticeable.

- General discussion

The experimental results showed that perceiving CRIs’ glossiness and roughness showed perceptual trends that differed from those of the real objects. In particular, glossiness was generally reduced by the colorimetric reproduction of the images, indicating that it is difficult to reproduce the perception of appearance. These results are consistent with a previous study [14], which found that appearance perception can differ between real stimuli and CRIs. This is thought to be due to the difference in the incident and reflected characteristics of light between the real object and the image. While real objects are associated with three-dimensional highlights and changes in brightness, images on display are flat and there are differences in visual information such as depth information from binocular vision and highlight overlaps due to disparity. Consequently, differences in the perception of glossiness and roughness can be expected.

The above results indicate that it is difficult to perceive gloss and roughness as equal to that of real objects by simply reproducing colorimetrically accurate images. There is also strong demand for an image reproduction method that can provide a perceptually equivalent appearance even in a single image.

### 2.4. Analysis of Physical Features of Objects That Affect Perceptual Alteration

The results of Experiment A indicate that color-managed CRIs may not be perceived as being as glossy or rough as the real ones. What, then, are the characteristics of the samples of which the perceptions changed significantly in comparison between the real object and the CRI? In this section, we clarify the physical characteristics of the samples with significantly changed perceptual values.

#### 2.4.1. Surface Roughness Measurement of a Real Object

The surface roughness of the real object stimulus was measured to obtain accurate optical information on the color and luminance distribution as well as its shape information. A 3D shape-measuring machine (VR-5100, Keyence Corp., Osaka, Japan) was used to measure the depth of the real stimulus surface with a resolution of 0.5 µm. Six physical quantities were obtained from the obtained shape data: arithmetical mean roughness (R_a_), maximum height (R_y_), ten-point mean roughness (R_z_), root mean square height (R_q_), skewness (R_sk_), and kurtosis (R_ku_) according to ISO 25,178 [18].

#### 2.4.2. Physical Features That Significantly Influence Glossiness and Roughness

Based on comparing the real object and the CRI in Experiment A, the samples with no significant difference in the evaluated glossiness and roughness values (effect size small or less) were defined as Group 1 (4 samples for glossiness and 25 samples for roughness), and the samples with significant difference were defined as Group 2 (63 samples for glossiness and 42 samples for roughness). Between these groups, Mann–Whitney U-tests were performed for the five gray-level co-occurrence matrix (GLCM) features (contrast, dissimilarity, homogeneity, ASM, and entropy) [19], color features (L* and chromaticity), and six surface roughness indices (R_a_, R_y_, R_z_, R_q_, R_sk_, and R_ku_), with *p* < 0.05 as the significance level. The GLCM indices were calculated by implementation in Python 3.8 according to the algorithm of the previous study [19], and each GLCM image feature was normalized to have a maximum value of 1.

- Glossiness

For glossiness, a significant difference in R_sk_ was confirmed. Specifically, when R_sk_ characteristics were compared between groups, a relationship of Group 1 > Group 2 was observed, with R_sk_ (Group 1 mean ± standard deviation, Group 2) = (−0.67 ± 1.25, −0.88 ± 0.72). Therefore, samples with low R_sk_ values (biased towards the convex side regarding object surface concavo-convex) have significantly reduced glossiness compared to the real object and the CRI, thus requiring more effort to reproduce glossiness in the image reproduction. Group 2 has a more negative R_sk_ value, indicating that the loss of glossiness in image reproduction occurs when the object surface is flat and has many smooth areas. The smooth glossiness that is widely distributed over the object surface, such as sheen gloss and contrast gloss (as identified by Hunter) [20], may affect the evaluation.

- Roughness

Significant differences were found in entropy, L*, and R_ku_ with regard to roughness perception (*p* < 0.05). Specifically, entropy (Group 1, Group 2) = 0.49 ± 0.18, 0.55 ± 0.15, L* (Group 1, Group 2) = 54.68 ± 25.17, 59.85 ± 24.85, and R_ku_ (Group 1, Group 2) = 1.60 ± 0.78, 1.90 ± 1.75. When comparing the characteristics between groups, all were significant with Group 1 < Group 2. This confirmed that roughness perception is sensitive to samples with complex surface textures comprising fine surface irregularities, as the entropy is lower for samples with simple and uniform object surfaces. 

## 3. Experiment B

Experiment B aimed to recover the perceptual transformation in image reproduction. A psychophysical experiment was conducted in which CRIs were processed to select images with perceptually equivalent glossiness and roughness, and their image features were analyzed.

### 3.1. Experimental Stimulus

The real stimuli are identical to those in Experiment A, while the image stimuli differ and are described in this section. To achieve perceptual image reproduction with glossiness and roughness perception equivalent to real objects, we first applied the gloss- and bumpiness-editing algorithms developed in our previous studies [21,22], which changed the appearance of the glossiness and roughness of the image. Using this method, we conducted an experiment to select images with gloss and roughness appearances equivalent to those of real stimuli from a set of images edited from CRIs and attempted to obtain perceptually equivalent reproduced images.

In the experiment, the real stimuli placed in the observation box were compared with the edited image stimuli shown on the display. One image was selected from the set of images whose glossiness and roughness were perceived to be equivalent to the real sample. Thirty-one images, comprising thirty processed images plus an unprocessed CRI, were used as the selection stimulus per trial. Otherwise described, when an unedited image was selected from the set of selected images, the perceived quality of the image was shown to be equivalent to the real object.

- Image stimulus for glossiness

**Figure 4:**
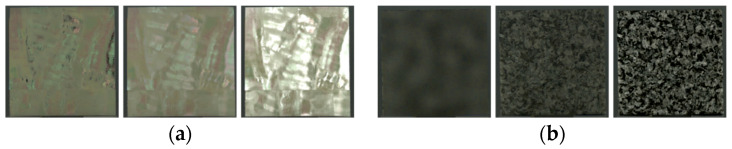
Example of image set (from left to right: suppression, original, enhancement). (**a**) Glossiness (white abalone, shell). (**b**) Roughness (granite, stone).

Prior to the experiment, a set of gloss-modified images was prepared. The gloss-editing method [21] edits the gloss component by emphasizing or suppressing gloss for the detected regions. Specifically, through gloss detection, high-lit areas in the image are pseudo-modulated by modulating the intensity information in the HSI color space for each of the image object’s pixel values. Figure 4a exemplifies an image with a modified gloss component. The gloss components of the CRIs for each stimulus were gradually edited to produce 30 gloss-edited images per stimulus.

- Image stimulus for roughness

The roughness-editing method [22] modulates the power spectrum of the low- and mid-frequency bands using the relationship between the image feature values and the bumpiness rating values of the image on the display, as modeled by the psychological rating experiments. The observer in this experiment is given the definition that roughness is an object’s surface unevenness, and bumpiness is also treated as roughness. Figure 4b shows an example of an image in which the bumpiness components that can affect roughness are changed. The frequency components of the CRIs for each stimulus were gradually edited, producing 30 roughness-edited images per stimulus.

### 3.2. Experimental Procedure

Experiments A and B were conducted in the same environment and employed the same procedural and observational protocols. The difference was that the image stimuli were not CRIs, but rather edited versions of CRIs. The observer changed the presented image stimulus using a keyboard and selected an image stimulus that he/she perceived to be equivalent in appearance to the real stimulus (perceptual reproduction image) from a set of previously prepared image stimuli. The observer was not informed how the image was changed by the keyboard operation. After the observer selected an image that produced an equivalent amount of perceived glossiness for a given real stimulus, the experimenter changed the stimulus and repeated the same rating. Sixty-seven stimuli were randomly presented, including material category, color, etc. First glossiness, then roughness was rated twice per stimulus, and one image per evaluation trial was selected for each glossiness and roughness perception that matched the real object.

### 3.3. Result

In Experiment B, observers selected images from the edited image set that they perceived to have the same glossiness and roughness as the real stimuli (hereafter referred to as perceptually reproduced images). The purpose of this experiment was to clarify which image features observers used to compare the glossiness/roughness perceptions with the real sample. To this end, we performed an analysis using GLCM features, a texture analysis method that uses image features extracted from 2D luminance images. The GLCM features of contrast, dissimilarity, homogeneity, and angular second moment (ASM) were used. These were useful for modeling physical glossiness in previous studies, especially contrast and entropy, which are suggested to be important indicators of glossiness and appearance [15]. Since real object features cannot be obtained without imaging, the image features of the CRIs used in Experiment A were analyzed as a surrogate. In Experiment B, the features of the average perceptually reproduced image selected by the observer were computed and the two were compared.

**Figure 5:**
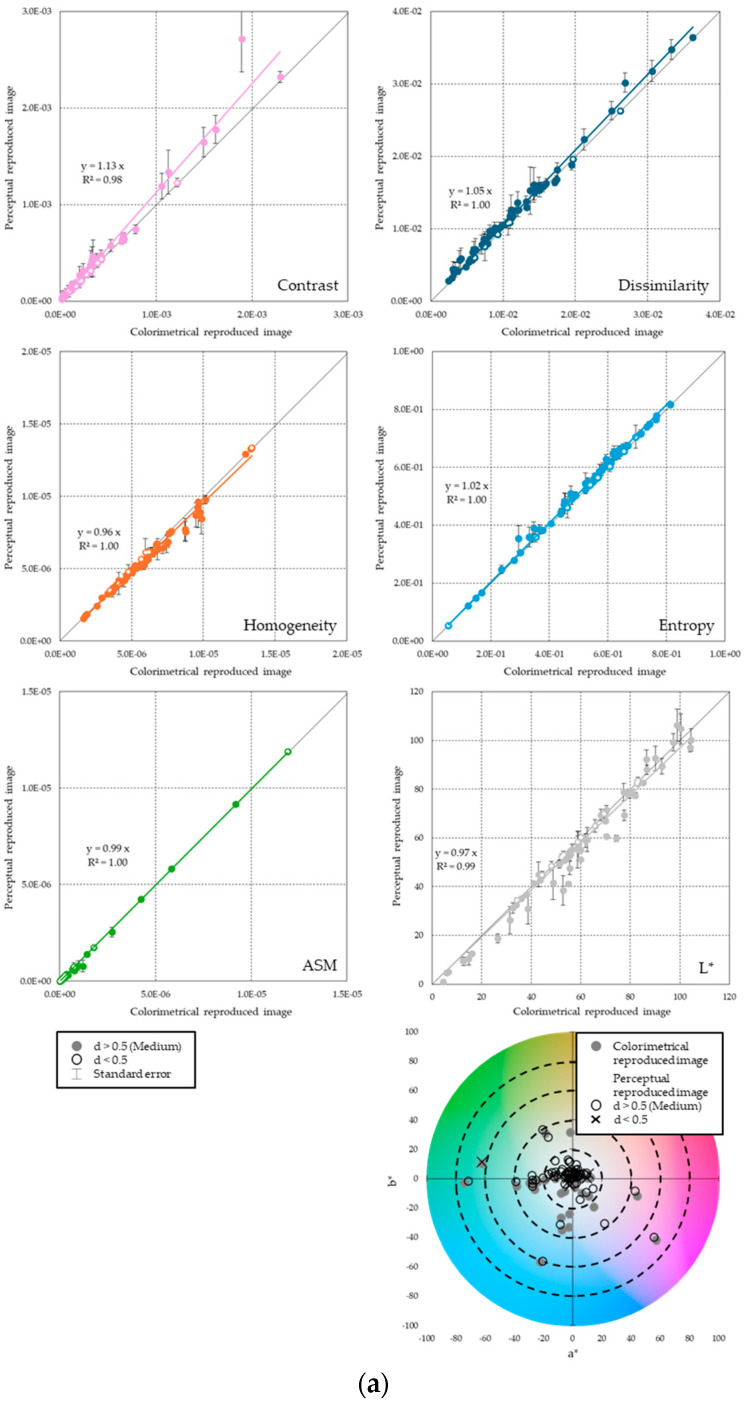

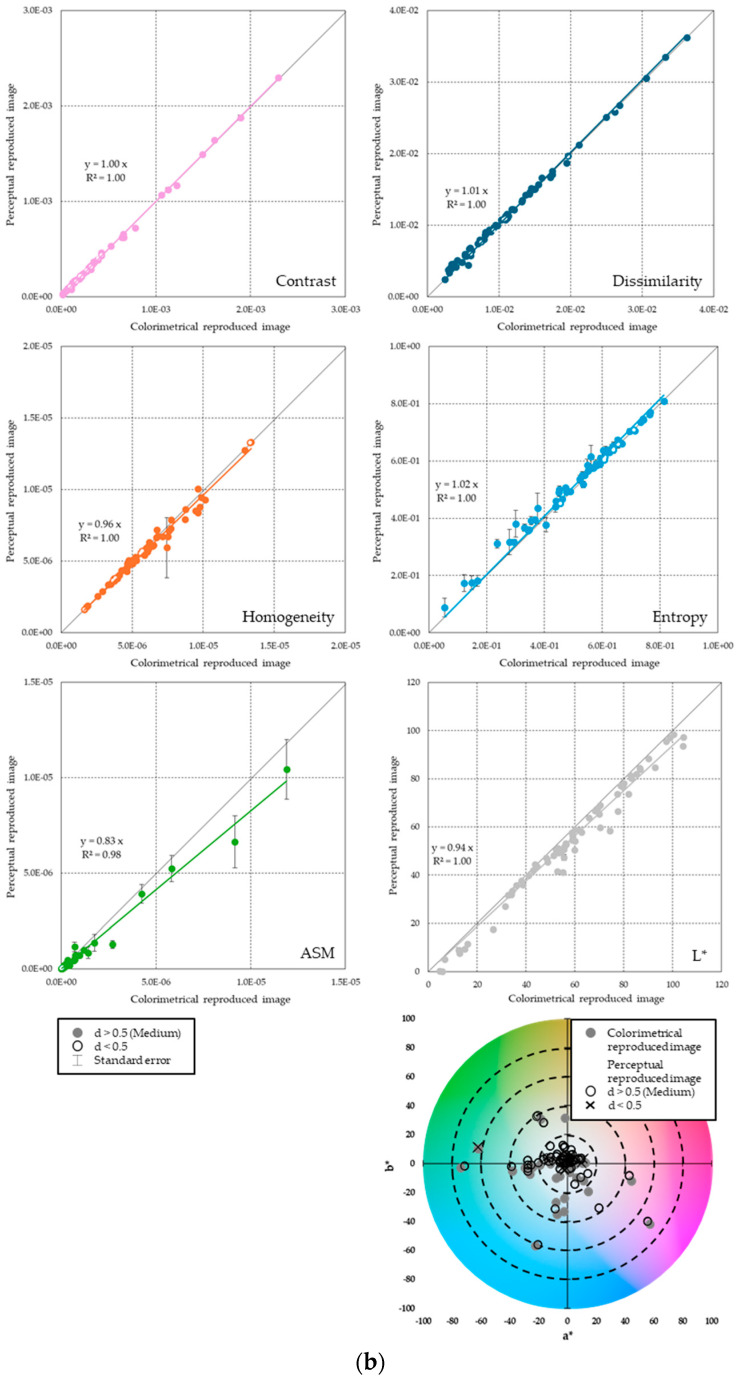
Image features for colorimetric and perceptually reproduced images (from top to bottom: contrast, dissimilarity, homogeneity, entropy, ASM, L*, and a*b* chromaticity). (**a**) Glossiness. (**b**) Roughness.

Figure 5 shows the results of the comparison of each feature. In the six scatter plots in the figure, the *x*-axis is the CRI and the *y*-axis is the perceptually reproduced image, and the contrast, dissimilarity, homogeneity, entropy, ASM, and L* features are plotted for each (the error bars are standard errors). For each plot, a filled circle indicates a sample with a significantly changed feature with an effect size of “medium” or greater (d > 0.5) in the CRI/perceptually reproduced image, and an open circle indicates a sample with no significant difference. Each scatterplot also shows the linear approximation equation (intercept = 0), the correlation coefficient, and the reference line (45-degree line, slope = 1) indicating the relationship between the CRI and the perceptually reproduced image. If the plot point is on the 45-degree line, then there is no significant difference in the feature values of the CRI and the perceptually reproduced image. If the plot point is above the 45-degree line, then it indicates that increasing the value of the feature improves perceptual reproducibility. Conversely, if the plot point is below the 45-degree line, then decreasing the value of the feature indicates that it is approaching the appearance of the real object. In the lower right of Figure 5, the a*b* chromaticity diagram is used to show the color information for each image.

- Glossiness

For glossiness (Figure 5a), a significant change was observed, particularly in contrast. The contrast has a long error bar, confirming a large variation in judgments between observers. On the other hand, the other indices generally follow the 45-degree line, and there is no difference in image features between the CRI and the perceptually reproduced images. It has been suggested that by increasing the contrast of the CRI by a factor of 1.13, glossiness can be reproduced that is perceptually equivalent to that of the real object. Prioritizing contrast modulation for gloss reproduction may affect the brightness and color of the image. However, the L* and a*b* chromaticity plots differed beyond color difference discrimination (ΔE*ab > 2.3) between the colorimetrically and perceptually reproduced images, suggesting that preserving color information has a low priority in glossiness perception. Considering the results of Figure 5a and the test results described in Section 2.4.2, the overall results indicate that for samples with the significantly reduced gloss sensations, image processing that increases the contrast of image features can perceptually reproduce the glossiness of the real object.

Our results are consistent with the findings of previous studies on gloss perception mechanisms [23] and modeling [15,24,25], indicating that contrast contributes significantly to gloss perception, even for specific CG stimuli and materials.

- Roughness

For roughness perception (Figure 5b), a significant change was observed, especially in the ASM. It was suggested that by suppressing the ASM of the CRI by a factor of 0.83, a roughness perception that is perceptually equivalent to that of the real object can be reproduced. No major changes were observed in any of the other characteristics that were aligned on the 45-degree line. Compared to the results of Experiment A, the roughness perception changed significantly in approximately 1/3 of the 67 stimuli samples. Therefore, it is believed that more accurate perceptual reproduction can be achieved by selectively applying appropriate image processing according to the physical characteristics of the target sample. Combined with those in Figure 4b, these results indicate that for samples with significantly lower roughness perception, image processing that reduces the ASM of image features can perceptually reproduce the roughness perception of the real object.

**Figure 6:**
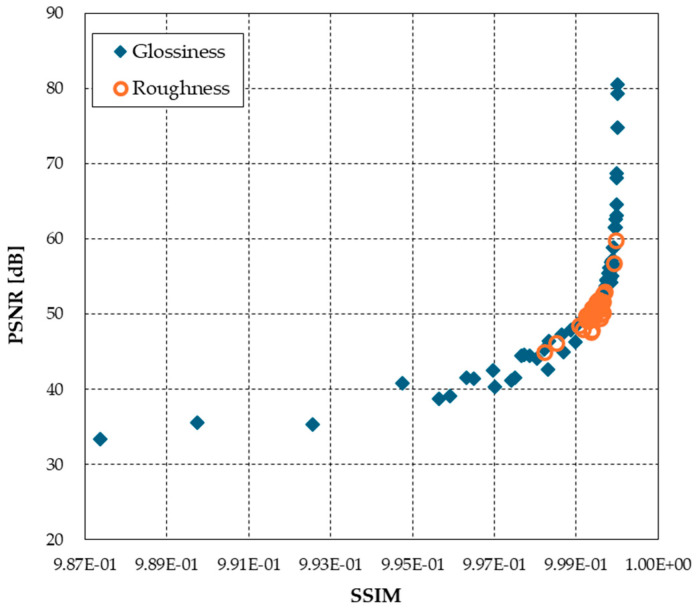
Image features of SSIM and PSNR between colorimetric and perceptually reproduced images.

Under the limited 2.5D experimental stimulus and illumination conditions used in this study, contrast for glossiness and ASM for roughness were found to be effective for perceptual reproduction; since the GLCM texture analysis was an index for the image stimulus surface, an image quality index for the entire image was also used for comparison. For reference, the values of structural similarity (SSIM) [26] and peak signal-to-noise ratio (PSNR) [27], which are common image quality evaluation indices, as the difference between CRI and average perceptually reproduced images, are shown in Figure 6. In this experiment, we tested these indices from the viewpoint of the degree to which the differences in images are recognizable, although this is different from the image quality degradation applications that use these indices. Normally, a PSNR of 30–40 dB and an SSIM of 0.90–0.98 are considered to be standard ranges where differences can be recognized when the image is enlarged. If the PSNR is above 40 dB and the SSIM is above 0.98, then the two images are indistinguishable. Although the SSIM for all images is 0.98, so the difference is imperceptible, there are some images below 40 dB in PSNR for glossiness. In order of decreasing PSNR, these image stimuli were white pottery (PSNR = 33.4), white canvas (35.4), gray drawing paper (35.6), white coral stone (38.7), and white abalone shell (39.2), all relatively bright color stimuli.

These results of this study show that even when color and resolution are reproduced with sufficient accuracy, appearance such as gloss and roughness, which are important indicators of an object’s surface, cannot be reproduced. This provides important insights for the development of hardware and software for imaging devices with HDR, a wider color gamut, and higher image resolution, which have long been goals in digital image processing, display technology, and material appearance modeling for industrial applications.

The image processing shown in this study according to the characteristics of real objects is not a realistic solution in today’s imaging technology and environment. However, it may be feasible for certain applications where objects can be measured, such as the digital archiving of works of art. It may also be feasible in the near future with the development of high-speed image rendering, such as real-time computer vision and computer graphics. In anticipation of these technological developments, this research should continue to explore ways to bring this technology to a more realistic point. In addition, a future challenge is to strive for image reproduction based on human-centered design that takes into account the diversity of human perception, which has been difficult to achieve with conventional approaches.

## 4. Conclusions

This study conducted two psychophysical experiments to analyze the physical features of real objects that affect glossiness and roughness alterations in image reproduction, as well as the image features necessary for their recovery. In total, 67 samples from 11 material categories, including metals and resins, were used as stimuli.

In Experiment A, we directly compared real images and CRIs to investigate changes in perceived gloss and roughness. The results showed that for most stimuli, the perceived glossiness was significantly reduced by imaging. In addition, both significant increases and decreases in perceived roughness were observed. In the analysis of physical features, the surface roughness information of the real surface showed that (1) the glossiness of samples with low skewness R_sk_ was significantly reduced by imaging, and (2) the roughness of samples with sharp surface irregularities and complex textures tended to be significantly reduced by imaging. These results suggest that although the glossiness and roughness of the real object and its CRI are perceived differently, it is possible to reproduce an image with perceptually equivalent glossiness and roughness by adjusting certain image features according to the characteristics of the real object during imaging.

Psychophysical Experiment B was conducted to directly compare the processed image with the real object intending to reproduce an image with a glossiness and roughness appearance perceptually equivalent to the real object. The experimental results were analyzed using GLCM features and color information, a type of texture analysis, and showed that contrast adjustment was effective as an image feature for recovering from altered glossiness. ASM adjustment was effective for recovering from altered roughness.

In this study, experimental analyses were performed on a limited number and variety of 2.5D experimental stimuli and illumination conditions. However, these samples are far from being representative of the abundance of real-world samples, and more diverse samples and conditions of illumination, observers, and image display need to be investigated and analyzed in detail to elucidate the universality and diversity of perceptual evaluation. Psychological approaches such as this study, which investigates the relationship between real-world stimuli and colorimetrically and perceptually reproduced images, have numerous challenges, which limit the extension of experimental conditions. However, if we take the approach of obtaining the number of observers or image stimuli by using appearance evaluation experiments based on no-reference images only, then the trade-off relationship is that the discussion is restricted to image feature values only, and there is no correspondence with the real world. In the future, it will be necessary to proceed with analysis by balancing high-quality offline experiments in a high-precision image reproduction environment and online experiments that ensure quantity even if the quality of image reproduction is low. We aim to develop more practical image-processing algorithms by expanding the experimental conditions through introducing 3D shape samples, the diversification of observers and material samples, and investigating other perceptual factors. In addition, more advanced image-processing methods will be investigated to analyze the perceptual mechanisms in detail.

## Data Availability

Data available on request due to restrictions.
